# Paradoxes of senolytics

**DOI:** 10.18632/aging.101750

**Published:** 2018-12-28

**Authors:** Mikhail V. Blagosklonny

**Affiliations:** 1 Cell Stress Biology, Roswell Park Cancer Institute, Buffalo, NY 14263, USA

**Keywords:** senescence, cancer, diseases, senolytic, mTOR, gerosuppressants

Senolytics are drugs that extend lifespan and delay some age-related diseases by killing senescent cells [1–4]. In fact, drug screens have identified a diverse group of drugs that are preferentially toxic to at least some senescent cells in some cellular models [2–9]. So far, however, their selectivity against senescent cells is modest and cell-type-specific [8–11]. Nevertheless, targeting senescent cells has been shown in animal models to prevent such age-related pathologies as emphysema [12], lung fibrosis [13–15], atherosclerosis [16,17], osteoporosis [18], osteoarthritis [19,20], renal disease [21], intervertebral disk pathology [2], hepatic steatosis [22] and other age-related conditions [4,7,18,23,24].

In this editorial commentary, I want to draw your attention to the paradoxes associated with senolytics, which argue against the dogma that says aging is a functional decline caused by molecular damage. This dogma predicts that senolytics should accelerate aging. If aging is caused by loss of function, then killing senescent cells would be expected to accelerate aging, given that dead cells have no functionality at all. Instead, however, senolytics slow aging, which highlights a contradiction in the prevailing dogma.

**Figure 1:**
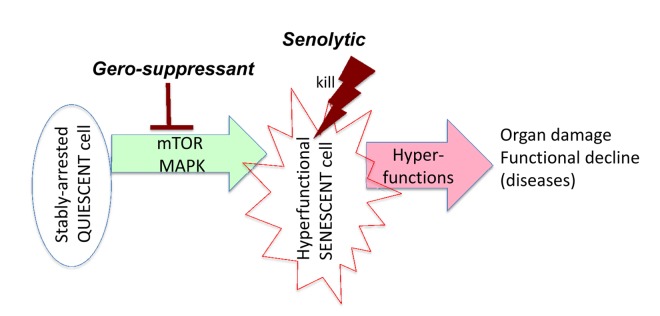
**Target of senolytics in the aging quasi-program.** In post-mitotic quiescent cells in an organism, growth-promoting effectors such as mTOR drive conversion to senescence. Hyperfunctional senescent cells activate other cells (including cells in distant organs), rendering them also hyperfunctional, which eventually leads to organ damage. This process manifests as functional decline, a terminal event secondary to initial hyperfunction. Senolytics such as ABT263 or 737 kill hyperfunctional senescent cells, preventing damage to organs. Gerosuppressants such as rapamycin suppress geroconversion and may decrease hyperfunction of already senescent cells, thereby slowing disease progression (not shown here in scheme).

The theory of hyperfunctional aging [25–32] addresses this paradox. Killing senescent cells is beneficial because senescent cells are hyperfunctional [33]. The hypersecretory phenotype or Senescence-Associated Secretory Phenotype (SASP) is the best-known example of universal hyperfunction [34–36]. Most such hyperfunctions are tissue-specific. For example, senescent beta cells overproduce insulin [37] and thus activate mTOR in hepatocytes, adipocytes and other cells, causing their hyperfunction, which in turn leads to metabolic syndrome (obesity, hypertension, hyperlipidemia and hyperglycemia) and is also a risk factor for cancer [38–40]. SASP, hyperinsulinemia and obesity, hypertension, hyperlipidemia and hyperglycemia are all examples of absolute hyperfunction (an increase in functionality). In comparison, relative hyperfunction is an insufficient decrease of unneeded function. For example, protein synthesis decreases with aging, but that decrease is not sufficient [30]. In analogy, a car moving on the highway at 65 mph is not “hyperfunctional.” But if the car were to exit the highway and enter a residential driveway at only 60 mph it would be “hyperfunctional,” and stopping that car would likely prevent damage to other objects. Similarly, killing hyperfunctional cells can prevent organismal damage. Senolytics eliminate hyperfunctional cells, which otherwise damage organs (Figure 1).

Senolytics should not be confused with gerosuppressants (Figure 1). Gerosuppressants, such as rapamycin, do not kill cells; they instead prevent cellular conversion to senescence (geroconversion) [33]. Rapamycin also slows disease progression by limiting the hyperfunction of senescent cells. Notably, some senolytics are also gerosuppressants. For example, inhibitors of MEK [41–43] or PI3K [2,41] are both gerosuppressants [41] and senolytics [2,42,43].

It may seem paradoxical that senolytics are anticancer drugs [44] because standard anticancer agents cause molecular damage. According to the hyperfunction theory [45], molecular damage does not cause aging. Although accumulation of molecular damage does happen and would destroy the organism eventually, no organism lives long enough for that to occur because TOR-driven (hyperfunctional) aging kills it first. If TOR-driven aging (i.e., aging as we currently know it) were abolished, then organisms would die from “post-aging syndrome” due to molecular damage (see Figure 8 in ref. [25].). Molecular damage contributes to some age-related diseases. But these diseases would arise even without molecular damage [45]. Molecular damage is essential for most types of cancer, but a senescent microenvironment [46] and overall organism aging (and associated diseases such as diabetes) also play roles [47], as does clonal selection for mTOR activation in cancer cells [48]. Importantly, molecular damage renders cancer cells robust and hyperfunctional. Cancer cells kill an organism not because molecular damage makes them weak; it is because the molecular damage makes them robust and hyperfunctional. If accumulation of molecular damage leads to immortalization and robustness, then aging cannot represent functional decline caused by molecular damage [48].

All senolytics, without exception, were initially investigated or specifically developed as anticancer drugs. But not all anticancer drugs are senolytics. Both senolytics and gerosuppressants belong to a very special subgroup of oncotargeted drugs [49]. Various pathways involving IGF-1, Ras, MEK, AMPK, TSC1/2, FOXO, PI3K, mTOR, S6K, and NFκB comprise a mTOR-related network and are involved in aging [49]. Oncoproteins promote aging, while tumor suppressors are gerosuppressors, which inhibit aging [48,50]. As depicted a decade ago (see Figure 3 in ref. [51]. and Figures 4 and 9 in ref. [25].), oncotargets are gerotargets that are also mTOR activators, while tumor and aging suppressors are mTOR inhibitors. In brief, geroconversion and oncogenic transformation are two sides of the same process [50]. Gerogenic oncogenes activate the mTOR pathway, driving geroconversion of cell cycle-arrested cells. When cell cycle control is disabled, they drive oncogenic transformation [48,50].

Many puzzles remain. For example, killing senescent adipocytes, macrophages or foam cells will slow diseases such as atherosclerosis and metabolic diseases, and killing senescent glial cells can prevent cognitive decline [23]. On the other hand, killing some senescent cell types may be counterproductive. For example, killing senescent beta cells may lead to diabetes [37], and killing of senescent hyperfunctional neurons in Alzheimer’s disease may have unpredictable consequences. Fortunately, senolytics are tissue-specific and only kill some types of senescent cells [8–11], which may make them safer.

To add further complication to the paradoxes associated with senolytics, it was shown that many detected p16/β-gal-positive cells are not senescent cells, but are instead hyperfunctional macrophages, which contribute to aging [52–54]. Notably, β-gal staining is a marker of hyperfunctional lysosomes [55]. A combination of markers, including mTOR targets, is needed to define senescence [33]. Some senolytics that target Bcl2 family proteins may theoretically kill leukemia/lymphoma cells. I hope to discuss these and other issues in a scheduled review “Senolytics, gerosuppressants and conventional life-extending drugs.”
